# Early postoperative measurement of fibroblast growth factor 23 predicts severe acute kidney injury in infants after cardiac surgery 

**DOI:** 10.5414/CN109359

**Published:** 2018-04-10

**Authors:** Oded Volovelsky, Katja M. Gist, Tara C. Terrell, Michael R. Bennett, David S. Cooper, Jeffrey A. Alten, Stuart L. Goldstein

**Affiliations:** 1 Center for Acute Care Nephrology, Cincinnati Children’s Hospital Medical Center, Cincinnati, OH,; 2 The Heart Institute, Department of Pediatrics, Children’s Hospital Colorado, University of Colorado Anschutz Medical Campus, Aurora, CO,; 3 The Heart Institute, Cincinnati Children’s Hospital Medical Center, Cincinnati, OH, and; 4 Department of Pediatrics, Section of Cardiac Critical Care, University of Alabama at Birmingham, Birmingham, AL, USA

**Keywords:** acute kidney injury, biomarkers, cardiac surgery, fibroblast growth factor 23, pediatrics

## Abstract

Aims: Acute kidney injury (AKI) occurs in 30 – 40% of children after cardiac surgery (CS) and is associated with poor prognosis. Fibroblast growth factor 23 (FGF23) is a bone-derived hormone with a pivotal role in phosphorus and vitamin D metabolism. We assessed FGF23 as an early marker for severe AKI (sAKI) in infants after CS. Materials and methods: Samples were previously collected in a multicenter observational study from children after CS. Serum FGF23 (n = 41) and urine AKI biomarker levels (n = 35) were assessed 4 – 8 hours after bypass. sAKI was defined as ≥ 100% rise in serum creatinine over baseline. Non-parametric and ROC analyses were used to evaluate the association between FGF23, urine AKI markers, and sAKI in the week after CS. Results: Serum FGF23, urine NGAL, and urine KIM1 were higher in sAKI patients. The AUC-ROC for urine NGAL (0.74, [0.49 – 0.99]), urine KIM1 (0.79, [0.68 – 0.98]), and serum FGF23 (0.74, [0.5 – 0.9]) showed fair prediction of sAKI. Conclusion: Early measurement of FGF23 has predictive ability in infants who develop sAKI after CS with cardiopulmonary bypass.

## Introduction 

The postoperative management of children with congenital heart disease has improved in recent decades with advancements in surgical techniques and cardiac intensive care. However, acute kidney injury (AKI) remains a source of significant morbidity in this population, with an incidence as high as 30 – 40% [1, 2, 3]. 

Infants are especially prone to AKI following cardiac surgery (CS), as the immaturity of the developing kidney makes infants more susceptible to injury. The use of cardiopulmonary bypass (CPB) increases the risk of renal injury in this population. AKI is associated with increased morbidity and mortality in patients after CS [1, 3, 4]. Therefore, early identification of AKI could potentially lead to improvement in outcomes for this susceptible and vulnerable population. 

The reliance on non-specific AKI markers such as creatinine and urine volumes has led to delayed diagnosis and treatment of AKI, and may lead to untreatable damage in these patients. Recent developments in the field including a standardized definition of AKI, early initiation of renal replacement therapy, and a better understanding of the pivotal role of fluid overload in AKI, have improved the care of these patients [5, 6, 7, 8]. Novel urine AKI-specific biomarkers have been identified, including neutrophil gelatinase-associated lipocalin (NGAL), interleukin-18 (IL-18), Kidney Injury Molecule-1 (KIM1) and liver-type fatty acid-binding protein (L-FABP). Postoperatively, these biomarkers have been shown to elevate prior to serum creatinine in development of AKI [9, 10, 11, 12]. The levels of these biomarkers are largely determined by damage to the kidney tissue, and therefore can be considered markers of structural kidney injury [13, 14, 15, 16]. 

The kidney is involved in the homeostasis of numerous biological systems, including hematopoiesis and bone and mineral metabolism. Functional AKI biomarkers should also be able to suggest derangements of kidney function in addition to tissue injury. A candidate for a functional marker of early AKI is fibroblast growth factor 23 (FGF23), a phosphaturic hormone which is produced by osteoblasts and osteocytes in the bone [17]. Increased FGF23 levels may be one of the earliest detectable markers of chronic kidney disease, prior to changes in calcium, phosphorous, or even parathyroid hormone (PTH) [18, 19]. Induction of AKI in mice by injection of folic acid led to rapid rise in FGF23 [20]. Small clinical studies in children have demonstrated an early increase in FGF23 levels in AKI [21, 22]. 

In this study, we explored the predictive performance of FGF23 for the development of severe AKI in infants with congenital heart disease who underwent CS with CPB. We compared FGF23 to other commonly used urine AKI biomarkers. We also examined whether the combination of FGF23 and other AKI-specific biomarkers can improve their predictive ability for the early identification of severe AKI. We hypothesized that FGF23, alone or in combination with other novel AKI biomarkers would identify development of AKI prior to a rise in serum creatinine. 

## Materials and methods 

### Design and participants 

We performed a post-hoc analysis of a prospective, multicenter cohort study of infants who underwent CS with CPB. Patients underwent CS from October 2013 to January 2015 at three pediatric medical centers: Cincinnati Children’s Hospital Medical Center (CCHMC), Children’s Hospital Colorado (CHC), and Children’s Hospital of Alabama (CHoA). The original study was conducted to determine if the level of AKI-specific urinary biomarkers predicted impaired milrinone clearance in infants after CS (data as yet unpublished, NCT01966237). Patients with congenital anomalies of the kidneys and urinary system were excluded as well as patients who needed extracorporeal life support (ECMO) in the perioperative period. The original and amended studies were approved by the institutional review boards at each institution, and written informed caregiver consent was obtained prior to enrolling patients in the original study. Each institutional board review (IRB) allowed for storage of biological samples for up to 5 years for additional analysis, contemplating their potential use for studies such as the current one. 

### Measurements 

Serum creatinine and electrolyte measurements were performed as part of standard clinical practice: levels were obtained preoperatively and daily for the first 3 days following surgery. AKI was defined by the pediatric modified RIFLE (pRIFLE) AKI criteria. Severe AKI (sAKI) was defined as either pRIFLE-I or -F (> 100% rise in serum creatinine over baseline) because these thresholds have been independently associated with morbidity and mortality in critically ill children, including those undergoing CS with CPB [3, 23]. 

Serum and urine samples were collected at scheduled intervals within the first 72 hours after the termination of CPB for studying milrinone pharmacokinetics and AKI biomarkers. We only utilized samples that were collected within 4 – 8 hours of termination of CPB in patients in ages 0 to 6 months for FGF23 measurement. Analysis of the samples was conducted at CCHMC. Blood and urine samples were centrifuged at 3,500 rpm and 4 °C for 10 – 15 minutes, and the serum extracted and immediately stored at –80 °C until quantification of milrinone was performed. The serum was thawed once for the current study in order to measure FGF23 level. The hormone level was measured in the central reference laboratory of CCHMC using the ELISA kit for human C-terminal FGF23 (Immutopics, Inc. San Clemente, CA, USA). Duplicates and control samples were used in the analysis. Serum levels may be reduced compared to plasm FGF23. 

Patient demographics and outcomes were previously collected by manual chart abstraction. Urine AKI biomarker data were utilized from the parent study, we did not re-analyze urine samples for the current study. Surgical complexity was categorized according to the Society of Thoracic Surgeons – European Association for Cardiothoracic Surgery, Congenital Heart Surgery (STAT) mortality categories [24]. All collected data from previous studies were de-identified, and each subject was given a unique study identification number which was used in all of the documents. Study data were collected and managed using REDCap (Research Electronic Data Capture) hosted at CCHMC. REDCap is a secure, web-based application designed to support data capture for research studies [25]. 

### Analytical and statistical methods 

All statistical analyses were performed using STATA (version 14, (College Station, TX, USA). The primary outcome was development of sAKI within the first 72 hours after CS. Comparative statistics were performed between subjects with vs. without sAKI. Categorical data were analyzed using χ^2^-tests. Skewed distributions were described with medians and interquartile ranges (IQR), and compared using the Mann-Whitney U-test. Receiver operator characteristic (ROC) curves were created for each biomarker to predict severe AKI, and the area under the curve (AUC) values were calculated for the different biomarkers and combination products of FGF23 and each novel AKI biomarker. A p-value of < 0.05 was considered to be statistically significant. 

## Results 

### Characteristics of cases and controls 

**Figure 1.**
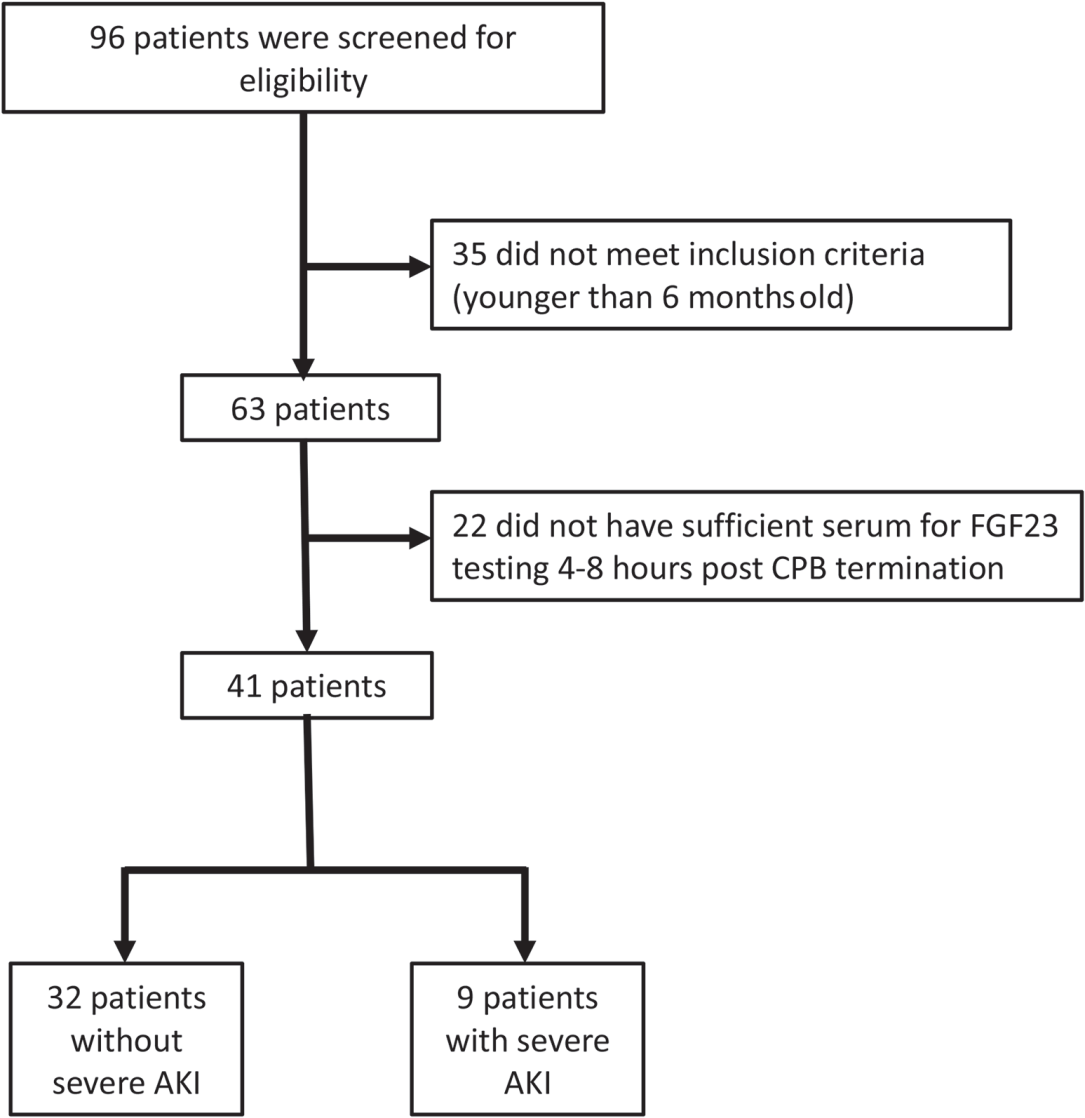
Screening, eligibility, and severe AKI status flow diagram for patient sample collection. AKI = acute kidney injury; CPB = cardiopulmonary bypass. Severe AKI is defined as a 100% rise in serum creatinine over baseline.

A total of 96 subjects across three medical centers were screened in the original study from October 2013 to January 2015. A total of 41 subjects (43%) were retained for analysis (Figure 1). Nine subjects (22%) in the current study group compared to 32 (78%) subjects in the screened group developed sAKI. We did not observe any differences in subjects with vs. without sAKI with respect to patient age, gender, race, STAT mortality score, and kidney function (Table 1). Although CPB time was 50% longer in subjects who developed severe AKI, this difference did not reach statistical significance. 

### Biomarkers and sAKI post CPB 

Biomarker concentrations were assessed between 4 and 8 hours after the termination of CPB; all 41 patients had serum remaining for FGF23 assessment, 35 of these patients had corresponding urine results for biomarkers. Median urine KIM1 (uKIM1) and urine NGAL (uNGAL) were higher in patients who developed sAKI (p < 0.05); median FGF23 was 60% higher in patients with sAKI (p = 0.07) (Table 2). 

### Biomarkers alone and in combination for prediction of sAKI 

Individual AUC-ROC for each biomarker was assessed from all available samples for that individual biomarker (Table 3). The AUC-ROCs [95% CI] for uNGAL (0.74, [0.49 – 0.99]), uKIM1 (0.79, [0.68 – 0.98]), and FGF23 (0.74, [0.5 – 0.9]) showed fair prediction for sAKI. The AUCs for the product of FGF23 and NGAL or KIM1 was performed using data from patients that had samples available for both FGF23 and the biomarker of interest (Table 3). Combination of FGF23 with uNGAL (0.80, [0.58 – 1]) and uKIM1 (0.82, [0.65 – 0.97]) increased the AUCs for each urinary biomarker, although this increase was not statistically significant. 

## Discussion 

In this multi-center post-hoc analysis of a prospective study, we examined 41 infants status post heart surgery, of which 9 subjects developed sAKI. The serum FGF23 concentration was 60% higher 4 – 8 hours after the termination of CPB in patients with severe AKI, similar to the previously studied biomarkers uNGAL and uKIM1. Interestingly, the levels of other biomarkers, including urine IL18 (uIL18) and LFABP (uLFABP), were not higher in subjects with sAKI. While the predictive performance of FGF23 for severe AKI was moderate (AUC = 0.74), combining FGF23 with uNGAL or uKIM1 increased performance slightly compared to individual biomarkers. 

FGF23 is involved in maintenance of normal phosphorous levels through reduction of phosphorous reabsorption in the renal proximal tubules via inhibition of the expression of sodium-phosphate co-transporters [26, 27, 28]. In addition, FGF23 inhibits the expression of the enzyme 25-hydroxyvitamin D-1-α hydroxylase, whilst stimulating the catabolic enzyme vitamin D-24-hydroxylase thereby leading to decreased intestinal absorption of phosphorous [29]. FGF23 levels rise in the very early stages of CKD due to mild interruptions of the homeostasis of calcium, phosphorous, calcitriol, and parathyroid hormone. A correlation between FGF23 and CKD progression has been demonstrated in infants as well [30]. However, the exact pathogenic role of FGF23 is still not well understood. 

Novel findings of research examining the role of FGF23 in AKI have opened a new window for using FGF23 as an AKI biomarker which is not solely dependent on the filtration capacity of the kidney. In animal models, FGF23 level increased abruptly after folic-acid induction of AKI and was independent of phosphorous and vitamin D levels [20]. The mechanism of the rapid increase is unclear, and may involve a release of stored hormone from the osteoblasts and osteroclasts or unknown sites, reduced degradation, or clearance by the kidneys [20, 22, 31]. In a pilot study in the same paper, the group has shown that in a small cohort of 14 patients who underwent CPB, an early rise in FGF23 was associated with a higher risk of severe AKI and poor postoperative outcomes [32]. The findings were confirmed in other adult studies. 

Two clinical studies on FGF23 in AKI after CS have been conducted in the pediatric population. The first was a nested case-control study comprised of 19 subjects of which 5 subjects developed AKI. The peak FGF23 from different sampling times after the surgery was correlated with the risk of AKI in that cohort [21]. The second comprised 32 subjects undergoing CS. The study examined the difference between the commonly used C-terminal FGF23 and its intact form and found that the level of FGF23’s intact form decreased to baseline earlier than the C-terminal form of FGF23 after AKI, possibly by increased proteolytic activity. The study included a wide age range, and the severity of AKI was mild due to the relatively low surgical complexity and short bypass time [22]. 

Our study has a number of relative strengths compared to previous pediatric CS studies of FGF23. It is the first multi-center study that includes three tertiary children’s hospitals from different regions of the US, comprising the largest pediatric study group with high surgical complexity. As a result, we observed a higher rate of sAKI. In addition, the study population includes mainly infants who have different FGF23 reference ranges than older populations. In addition, cardiac surgeries in infants are usually more complex than in children and adolescents. This is also the first pediatric research study which compares FGF23 to other novel urinary AKI biomarkers in the same patients [16]. 

Several limitations in our study should be noted. We measured FGF23 in serum samples which were previously collected, and previously thawed. Similar to other studies, we measured only the C-terminal FGF23 and not the intact form of the hormone. Although hypoxemia can affect the FGF23 level, the preoperative oxygen level is not available. Urine output was not used as a criterion for AKI as the data were already collected in a previous study. We only used one sample of serum and not a series of samples. As this is a post-hoc study the power of the research cannot be sufficient to rule out differences due to the lack of the ability to prospectively plan the study sample size. 

In conclusion, among infants undergoing CS with CPB, our data suggests that early measurement of FGF23 may have some predictive ability, but larger studies are required to make this determination. Given the inherent limitations of our post-hoc analysis, we recognize that prospective studies are required to validate the associations we observed. 

## Funding 

This study was supported by grant funding from the E.W. Thrasher Foundation (Katja Gist). 

## Conflict of interest 

No potential conflict of interest was reported by the authors. 

**Table 1.** Patient characteristics.

Characteristic	All (n = 41)	Without severe AKI (n = 32)	Severe AKI (n = 9)	p-value
Age (days)	130 (57, 149)	127 (56, 145)	137 (59, 155)	0.75
Males (%)	27 (66)	20 (62)	7 (78)	0.39
Weight (kg)	5.2 (4.1, 5.9)	4.8 (4.2, 6)	5.6 (4.3, 6)	0.63
Caucasian	34 (83)	25 (78)	9 (100)	0.31
STAT mortality score	3 (2, 4)	3 (2, 4)	3 (2, 4)	0.85
Creatinine level (mg/dL)	0.32 (0.30, 0.40)	0.33 (0.30, 0.40)	0.33 (0.30, 0.40)	0.40
BUN (mg/dL)	11 (8, 15)	11.5 (8, 16)	10 (9, 11)	0.56
GFR (mL/min/1.73m^2^)	74 (57, 83)	73 (57, 81)	80 (65, 106)	0.33
CPB time (min)	111 (74.5, 172)	111 (69, 157)	113 (83, 291)	0.31

Values are presented as n (%) or median (interquartile range [IQR], 25^th^ – 75^th^ percentile). Mann-Whitney and χ^2^-tests were used for continuous and categorical variables, respectively. GFR was calculated using Schwartz formula (k = 0.413). Severe AKI was defined as stage I or R based on RIFLE criteria. AKI = acute kidney injury; BUN = blood urea nitrogen. CPB = cardiopulmonary bypass; GFR = glomerular filtration rate; STAT = Society of Thoracic Surgeons-European Association of Cardio-Thoracic Surgery Congenital Heart Surgery Mortality.

**Table 2.** Postoperative variables.

Characteristic	All	Without severe AKI	Severe AKI	p-value
uKIM1 (mg/mL)^a^	191 (100, 344)	146 (93, 228)	550 (269, 588)	0.006
uNGAL (ng/mL)^a^	149 (28, 471)	135 (24, 324)	589 (221, 1,071)	0.05
uIL18 (pg/mL)^a^	526 (157, 1,158)	534 (157, 1,088)	953 (231, 1,387)	0.64
uLFABP (mg/mL)^a^	83 (37, 271)	109 (68, 462)	164 (77, 271)	0.5
Serum FGF23 (RU/mL)^b^	806 (365, 1,325)	684 (244, 1,246)	1,092 (637, 1,705)	0.07

^a^35 patients total – 28 without severe AKI, 7 with severe AKI; ^b^41 patients total – 32 without severe AKI, 9 with severe AKI. Values are presented as median (interquartile range [IQR], 25^th^ – 75^th^ percentile). Mann-Whitney test was used to compare the group without severe AKI to the group with severe AKI. A p-value for global comparisons among groups by Kruskal-Wallis. Severe AKI was defined as stage I or R based on RIFLE criteria. Biomarker levels were obtained 4 – 8 hours after cardiopulmonary bypass. AKI = acute kidney injury; FGF23 = fibroblast growth factor 23; uIL18 = urine interleukin 18; uNGAL = urine neutrophil gelatinase-associated lipocalin; uKIM1 = urine kidney injury molecule-1; uLFABP = urine liver-type fatty acid-binding protein.

**Table 3.** Receiver operating characteristic analyses of the prediction of severe acute kidney injury.

Marker	AUC-ROC (95% CI)
uNGAL (ng/mL)	0.74 (0.49 – 0.99)
uKIM1 (pg/mL)	0.79 (0.68 – 0.98)
uIL18 (pg/mL)	0.56 (0.29 – 0.83)
uLFABP (mg/mL)	0.59 (0.35 – 0.82)
FGF23 (RU/mL)	0.74 (0.50 – 0.90)
FGF23 × uKIM1	0.82 (0.65 – 0.97)
FGF23 × uNGAL	0.80 (0.58 – 1.0)

AUC = area under curve; FGF23 = fibroblast growth factor 23; uNGAL = neutrophil gelatinase-associated lipocalin; uKIM1 = kidney injury molecule-1; IL-18 = interleukin-18; uLFABP = liver-type fatty acid-binding protein; ROC = receiver operating characteristic; SEM = standard error of mean.
